# Research Advances in Critical Care: Targeting Patients' Physiological and Psychological Outcomes

**DOI:** 10.1155/2015/283067

**Published:** 2015-10-26

**Authors:** Elizabeth Papathanassoglou, Ged Williams, Julie Benbenishty

**Affiliations:** ^1^Faculty of Nursing, Edmonton Clinic Health Academy (ECHA), University of Alberta, 11405-87th Avenue, Edmonton, AB, Canada T6G 1C9; ^2^Department of Nursing, Cyprus University of Technology, 15 Vragadinou Street, 3041 Limassol, Cyprus; ^3^Griffith University, 170 Kessels Road, Nathan, QLD 4111, Australia; ^4^Abu Dhabi Health Service Company, P.O. Box 109090, Abu Dhabi, UAE; ^5^Hadassah Hebrew University Medical Center, Kiryat Hadassah, P.O. Box 12000, 91120 Jerusalem, Israel

Critical illness is an immensely complex entity, affecting simultaneously cellular, neurohormonal, immune, endothelial, and organ system responses and at the same time entailing intricate psychosocial and cognitive correlates [1]. Outcomes in critical illness largely depend on the interplay among these response systems, rather than on any specific factor. Hence, critical illness and its consequent complications continue to challenge clinicians throughout the world.

Recent developments in critical care show that collaborative interdisciplinary interventions targeting patients' needs as well as focusing on care have the potential to improve survival outcomes, as well as longer-term physiological and psychological outcomes in many critically ill individuals [2]. These interventions include approaches such as infection control, management of sedation, agitation, and pain, body positioning and early progressive mobilization, palliative care, family presence and visitation, management of anxiety, and nonpharmacologic interventions to promote sleep and relaxation, and they encompass the principles of bundled care and interprofessional collaboration. Moreover, recently, the impact of organizational factors on patients' outcomes and survival has been highlighted [1].

In the present issue a wide range of research topics is depicted that reflects the importance of interdisciplinary efforts for the improvement of critically ill patients' outcomes. Studies span numerous countries with the majority having been conducted in Europe.

Two studies address practice-related issues and stress the importance of clinical competence for critical illness outcomes and one study addresses organizational factors and points to the necessity of focusing on information management and collaboration. Moreover three literature reviews on the impact of clinical protocols on patients' outcomes, including pain assessment, sedation, and adrenergic blocking, are included. Articles mainly address the acute phase of illness; however, a study from Denmark explores the association of delirium while in the intensive care unit (ICU) with posttraumatic stress disorder symptomatology. Most of the studies are primary research papers, whilst, in three, authors systematically review evidence on recent developments of practice.

R.-L. Lakanmaa et al. conducted a cross-sectional survey of self-perceived competence among Finish intensive care nurses to find that the majority of nurses perceived their competence as good on a 5-point scale ranging from poor to excellent, despite an overall low experience. Remarkably, the factor explaining the greater percentage of variation in competence ratings was respondents' experience of nursing autonomy which emphasizes the importance of a healthy work environment in critical care. Indeed, previous studies have highlighted the interdependence between autonomy and collaboration in critical care [3]. E. B. Sendin et al. addressed proper use of alarms with regard to monitoring and mechanical ventilation, which is a topic poorly addressed in research literature. They employed a prospective observational study in a neonatal intensive care unit (ICU) in Spain to explore proper use of equipment safety mechanisms. The overall rate of appropriate use of equipment was low (33.68%), irrespective to patient characteristics; and it associated with late night and weekend shifts as well as with higher occupancy, at which cases improved usage was noted. The authors point to the importance of identifying and using appropriate alarms for patient safety, and although not addressed in their design they provide a discussion of adverse outcomes potentially linked to improper use of alarms. The incidence of admission and discharge delays and associated organizational factors were the focus of a systematic review by L.-M. Peltonen et al. The study highlights both the magnitude of the problem, with delays being common involving ICU admission (38%) and discharge (22–67%) processes, and the lack of explanatory research addressing these delays. The authors emphasize the need to revisit the design of care processes by focusing on information management and coordination between units and interdisciplinary teams.

In a systematic review of randomized clinical trials comparing dexmedetomidine to other sedation strategies with regard to the risk of delirium, dexmedetomidine was suggested to allow for avoidance of deep sedation and use of benzodiazepines, factors both associated with developing delirium; however, conclusive results as to the incidence of delirium were not possible, therefore warranting further research.

E. Georgiou et al. conducted a systematic review to explore the impact of pain assessment as an independent intervention on critically ill patients' outcomes. Implementation of systematic approaches to pain assessment appears to be associated with higher frequency of written documentation of pain and more efficient decisions for pain management. Moreover, the studies provided evidence for associations with decreased pain intensity, duration of mechanical ventilation, length of ICU stay, mortality, adverse events, and complications. The authors emphasize the need for more randomized clinical trials to address this topic. Outcomes related to beta blocking in patients with septic shock are the focus of the critical literature review by P. Pemberton et al. The study raises issues regarding selection of beta-blocking agents and patients most likely to benefit as well as timing of treatment.

The papers in this special issue highlight the breadth of issues related to the critical illness trajectory and the diversity of practice approaches to improve outcomes and they raise stimulating questions for future exploration. The papers emphasize different means to improve outcomes spanning a wide spectrum of roles and interventions from the organizational and educational to pharmacological means and clinical assessment. Factors that appear to be central to all these approaches are sound patient assessment and team collaboration. We hope that readers will enjoy and be inspired by these papers to pursue future research work and practice development in their specific field of interest.

*Elizabeth Papathanassoglou* *Ged Williams* *Julie Benbenishty*
